# Supplementary material for: The influence of trace metal supplementation on the presence of ceftriaxone resistance in Enterobacteriaceae in the gastrointestinal tract of dairy cattle
Source: Microbiol Spectr. 2025 Feb 19;13(4):e01090-24. doi: 10.1128/spectrum.01090-24 (PMC11960429; doi:10.1128/spectrum.01090-24)
Supplement: Table S1 — MIC concentration determination. [file spectrum.01090-24-s0001.docx]

| Supplemental Table 1: Final minimum inhibitory concentration (MIC) of copper and zinc as determined using Mueller-Hinton broth microdilutions and agar plating | | | | | |
| --- | --- | --- | --- | --- | --- |
| Isolates | Microdilutions | | Agar | |  |
|  | MIC CuSO4 (mM) | MIC ZnCl2 (mM) | MIC CuSO4 (mM) | MIC ZnCl2 (mM) |  |
| *E.coli 25922* | 16 | 2 | 16 | 2 |  |
| *E. faecalis 2128* | 24 | 6 | 24 | 6 |  |
| *Lelliottia amnigena AN-Abat-3L-A01* | 24 | 4 | 24 | 8-Jun |  |
| *AN1-R2-P14-01* | 24 | 4 | 24 | 6 |  |
| *AN1-R1-P01-01* | 48 | >8 | 32-48 | >8 |  |
